# Effect of Bitter Compounds on the Expression of Bitter Taste Receptor T2R7 Downstream Signaling Effectors in *cT2R7*/pDisplay-G*α*16/gust44/pcDNA3.1 (+) Cells

**DOI:** 10.1155/2019/6301915

**Published:** 2019-10-31

**Authors:** Yuan Su, Hang Jie, Qing Zhu, Xiaoling Zhao, Yan Wang, Huadong Yin, Shailendra Kumar Mishra, Diyan Li

**Affiliations:** ^1^Farm Animal Genetic Resources Exploration and Innovation Key Laboratory of Sichuan Province, Sichuan Agricultural University, Chengdu 611130, China; ^2^Chongqing Engineering Technology Research Center for GAP of Genuine Medicinal Materials, Chongqing Institute of Medicinal Plant Cultivation, Nanchuan, Chongqing 404100, China

## Abstract

Bitterness is an important taste sensation for chickens, which provides useful sensory information for acquisition and selection of diet, and warns them against ingestion of potentially harmful and noxious substances in nature. Bitter taste receptors (T2Rs) mediate the recognition of bitter compounds belonging to a family of proteins known as G-protein coupled receptors. The aim of this study was to identify and evaluate the expression of *T2R7* in chicken tongue tissue and construct *cT2R7-1* and *cT2R7-2-*expressing HEK-293T cells to access the expression of *PLCβ2* and *ITPR3* after exposure with different concentrations of the bitter compounds. Using real-time PCR, we show that the relative expression level of *T2R7* mRNA in 5, 1, 0.1, and 10^−3^ mM of camphor and erythromycin solutions and 5 mM of chlorpheniramine maleate solutions was significantly higher than that in 50 mM KCL solutions. We confirmed that the bitter taste receptor T2R7 and downstream signaling effectors are sensitive to different concentrations of bitter compounds. Moreover, T2R7-1 (corresponding to the unique haplotype of the Tibetan chicken) had higher sensitivity to bitter compounds compared with that of T2R7-2 (corresponding to the unique haplotype of the Jiuyuan black-chicken). These results provide great significance of taste response on dietary intake to improve chicken feeding efficiency in poultry production and have certain reference value for future taste research in other bird species.

## 1. Introduction

Hitherto, there are five widely known basic tastes that stimulate and are perceived by taste buds—sweet, umami, sour, bitter, and salty [1, 2]. Among them, bitter taste is an important natural defense providing useful sensory information for animals to ensure them ingestion of potentially healthy feeds rather than harmful/toxic substances [3–5]. Identification of bitter compounds is mediated by the type 2 taste receptors (T2Rs), which belong to G-protein-coupled superfamily [6, 7]. Interestingly, bitter taste receptors vary among species: a much smaller T2Rs gene repertoire exists in birds (range from 1 in the domestic pigeon to 12 in the bar-tailed trogon) in comparison to 10–69 in mammals [8, 9], and chickens (*Gallus gallus domesticus*) have only three functional bitter taste receptors comprising T2R1, T2R2, and T2R7 [10–12]. Intriguingly, white-throated sparrows (*Zonotrichia albicollis*) were found to have 18 putatively functional T2Rs, indicating their sensitivity to wider range of bitter substances [13].

In recent years, tremendous progress has been made to elucidate different taste senses mediated by taste receptors located in oral cavity. In birds, bitter taste sensitivity varies within and between species. For instance, laying hens and turkeys have a high response to glucosinolates than broilers [14]. A behavioral study showed that T2R1 is highly sensitive to dextromethorphan (Dex) and diphenidol (Dip) agonists affecting the behavioral responses to bitterness in chicken [15]. Recently, 6-methoxyflavanone has been identified as an antagonist for functional bitter receptors (T2R1 and T2R7) of oral epithelial cells through Ca^2+^ imaging technology [16]. Moreover, cell-based assay showed high expression levels of *T2R7* and *T2R1* in Vimentin^−ve^ taste bud cells, indicating that T2R7 and T2R1 mediate the functional bitter and umami sensation in chicken, respectively [17]. Furthermore, previous studies had reported that chickens and cockatiels (*Nymphicus hollandicus*) are similar to humans in their sensitivity to quinine, but superior to many other mammals [18, 19]. Additionally, chickens are more responsive to glucosinolates than ruminants [20]. Recently, hen protein hydrolysate (HPH) peptides are screened as the blockers of T2R4, T2R7, and T2R14 in HEK293T cell-based assay [21].

Compared with mammals, birds possess inferior taste acuity, mainly manifested as insufficient chewing ability, relatively small number of taste buds, and low saliva secretion [22]. Although T2Rs are sensitive to several bitter substances, various T2Rs exhibit different response towards the same bitter compounds (broadly “tuned”). Recently, in terms of bitterness, after screening 46 different bitter compounds, 17 bitter compounds have been proved to activate T2R7 in chicken [23]. Interestingly, one report showed a positive correlation between the number of taste buds in different breeds of chicken and their bitterness sensitivity and that the layers appear to have a high sensitivity to quinine hydrochloride than those of broilers [24]. Previous studies have shown that the bitter taste receptor gene family (*T2Rs*) and their downstream genes (*α-gustducin*, *PLCβ2*, and *TRPM5*) are expressed in the chicken's gustatory and extraoral gastrointestinal tract (GIT) tissues [25, 26].

By examining quinine analogs through integrated in silico and *in vivo* approach, three new T2R1 agonists (epiquinidine, ethylhydrocupreine, and quinidine) are identified that contribute to increase our understanding of bitter perception in chickens [27, 28]. In chickens, 2-choice test has been established to detect taste threshold for bitter, umami, and sweet tastes which have the potential to affect feed and water consumption [29]. Cheled-shoval et al. [29] found that chickens had similar thresholds to humans for quinine (bitter) but were less sensitive to sucrose and monosodium glutamate. In chickens, a study on bitter taste system demonstrated that behavioral (*in vivo*) thresholds are similar or up to two orders of magnitude higher than the heterologous (*in vitro*) ones [30].

In chickens, recent studies have specifically identified bitter taste receptors activated by various bitter compounds [15, 23]. In our previous study on *T2R7* gene, we found that haplotype HE1 corresponding to the Tibetan chicken was positively associated with high-altitude adaptation, while haplotype HE4 corresponding to the Jiuyuan black-chicken showed a negative correlation with high-altitude adaptation [31]. It shows that the elucidation of the chicken bitter taste sensitivity to different bitter compounds is extremely important in the development of chicken feed efficiency in poultry production, but the bitterness sensitivity mediated molecular mechanism is still to be explored. Therefore, in this study, we focused on the investigation of the influence of different bitter compounds on the expression of bitter taste receptor T2R7 and downstream signaling effectors in *cT2R7*/pDisplay-G*α*16/gust44/pcDNA3.1 (+) cells.

## 2. Materials and Methods

### 2.1. Chemicals

Camphor, chlorpheniramine maleate, erythromycin, benzoin, chloramphenicol, quinine, parthenolide, and KCL were purchased from Sangon Biotech Co. Ltd. Chengdu, China. For individual and cell stimulation experiments, KCL was dissolved in ultrapure water to prepare a 50 mM KCL solution. Other compounds were dissolved in 50 mM KCL solution to make a 5 mM stock solution, and the 5 mM stock solution was diluted with 50 mM KCL solution to make 1, 0.1, 10^−3^, and 10^−5^ mM solutions. These solutions of bitter chemical compounds were stored at −20°C until use.

### 2.2. Animals and Individual Tests

A total of 108 Jiuyuan black-chickens (54 males and 54 females) at 120 days of age were used in this study. The same feeding management and regimens were provided to all chickens. This research was conducted under the Welfare and Management of Animals Act and approved by the Committee on the Care and Use of Laboratory Animals of the State-Level Animal Experimental Teaching Demonstration Centre of Sichuan Agricultural University (Approval ID: Decree number S20160906). Experimental procedures were performed in accordance with the Regulations for the Administration of Affairs Concerning Experimental Animals (China, 1988) for animal experiments. All efforts were made to minimize the suffering of the chickens, and no animal was injured during capture. Each independent experiment was performed in triplicate, and all three treated chickens had a similar weight. Briefly, the chemical compound test solutions (1 mL) were given with water to randomly selected chickens during the experimental period. After the exposure to the chemical compound for 24 hours, the whole tongue tissue samples were rapidly collected, frozen in liquid nitrogen, and stored at −80°C until use.

### 2.3. Preparation of Chicken *T2R7-1* and *T2R7-2* Plasmid Constructs

We fixed the point mutations using site-directed mutagenesis kit (Sangon Biotech Co. Ltd. Chengdu, China) to obtain haplotype HE1 (the unique haplotype of the Tibetan chicken) and haplotype HE4 (the unique haplotype of the Jiuyuan black-chicken), which were identified in our previous research [31]. The mutations identified in cDNA sequences of *T2R7-1* (haplotype HE1, KT377158) and *T2R7-2* (haplotype HE4, KT377161) and corresponding amino acid residues are shown in Table 1. The PCR products of the *T2R7-1 and T2R7-2* ORF were subcloned into the pDisplay™ (Invitrogen) mammalian expression vector by using In-Fusion HD Cloning Kit (TaKaRa Biotech Corporation, Dalian, China). The endotoxin-free recombinant plasmids *cT2R7-1*/pDisplay and *cT2R7-2*/pDisplay were extracted using the E.Z.N.A. Endo-Free Plasmid Mini Kit I (Omega Bio-tek®) following the manufacturer's protocol. G*α*16/gust44/pcDNA3.1 (+) was purchased from Sangon Biotech Co. Ltd. Chengdu, China.

### 2.4. Cell Cultures

Human embryonic kidney-derived 293T (HEK-293T) cells were kindly donated by Pro. Jiwen Wang's waterfowl research group (Sichuan Agricultural University). HEK-293T cells were grown in Dulbecco's Modified Eagle Medium (DMEM) supplemented with 10% FBS at 37°C under 5% CO_2_ overnight. Briefly, when the HEK-293T cells were at ∼80% confluence, the cells were cotransfected with G*α*16/gust44/pcDNA3.1 (+) and *cT2R7-1*/pDisplay or *cT2R7-2*/pDisplay (the ratio, *T2R7*-pDisplay: *Ga16gust44*-pcDNA3.1 (+) = 4 : 1) in a six-well plate using LipofectAMINE2000 (Invitrogen). About 24 hours after transfection, the cells were stimulated with 0.1 mM and 10^−3^ mM chemical compound test solutions (described above) for 1 hour. Total RNA was collected from the cells and used to evaluate the relative mRNA expression of *PLCβ2* and *ITPR3*.

### 2.5. RNA Extraction and Real-Time PCR

Total RNA was extracted using RNAiso Plus (TaKaRa Biotech Corporation, Dalian, China) kit and reverse transcription was performed using the PrimeScript™ RT Reagent Kit with gDNA Eraser (TaKaRa Biotech Corporation, Dalian, China). Primers for quantitative real-time PCR were designed using Primer v5.0, listed in Table 2. PCR mixture consisted of 1 *μ*L of cDNA, 0.8 *μ*L of 10 pmol/*μ*L each primer, 12.5 *μ*L of SYBR1Premix Ex Taq™ II (TaKaRa Biotech Corporation, Dalian, China), and 9.9 *μ*L of double-distilled H_2_O in a final reaction volume of 25 *μ*L. Thermocycling parameters were 95°C for 2 min followed by 40 cycles of 95°C for 5 s, the annealing temperature for 30 s, and 60°C for 30 s.

### 2.6. Statistical Analysis

Statistical analysis was performed using one-way ANOVA with the SAS v9.0 statistical software package (SAS Institute Inc., Cary, NC, USA) to test the differences among treatments. All data are presented as the mean ± standard error (SE), and *P* value < 0.05 is considered statistically significant.

## 3. Results

### 3.1. Effect of Different Bitter Compounds on the Expression of *T2R7* mRNA in Tongue Tissue of Chicken

**Figure 1:**
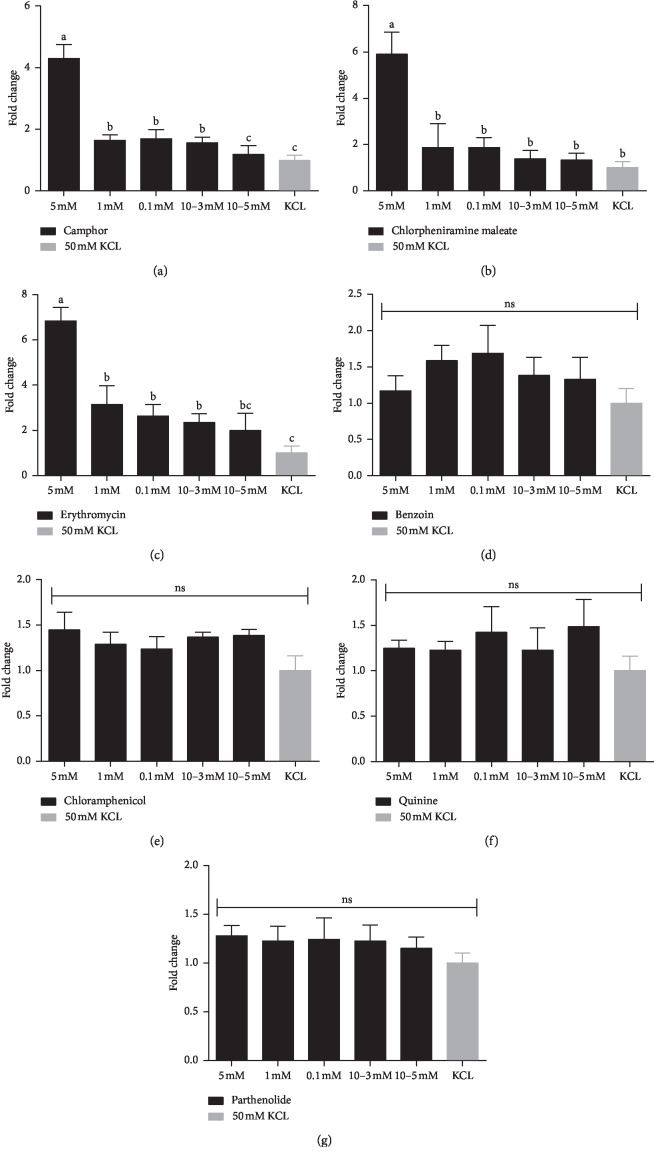
Relative expression levels of *T2R7* mRNA in tongue tissue of chicken stimulated by different concentrations of (a) camphor, (b) chlorpheniramine maleate, (c) erythromycin, (d) benzoin, (e) chloramphenicol, (f) quinine, and (g) parthenolide, as determined by real-time PCR. The relative gene expression was measured by qPCR and normalized to GAPDH, and a 50 mM KCL solution was the negative control. Each bar represents the mean ± SE of the results from 2 to 3 independent experiments performed in triplicate. Different small letters on the bar indicate significant differences at *P* < 0.05.

To investigate the effect of various bitter compounds in chicken, tongue tissues of chicken were stimulated with different chemical compound solutions. The relative fold changes in *T2R7* mRNA expression from the RT-PCR experiments are summarized in Figure 1. The effect of different concentrations of camphor solution on the *T2R7* mRNA expression was significant (*P* < 0.05) (Figure 1(a)). In camphor solution, the relative fold change of 5 mM was significantly higher than that of the other concentrations (*P* < 0.05). Camphor solutions of 1, 0.1, and 10^−3^ mM concentrations had significantly higher fold changes than solutions of 10^−5^ mM (*P* < 0.05), but no significant difference was found among the three camphor solutions (*P* > 0.05). Moreover, there was no significant difference detected in *T2R7* mRNA expression between 10^−5^ mM camphor solution and 50 mM KCL solution (*P* > 0.05). The effects of chlorpheniramine maleate solution with different concentrations on the expression of *T2R7* mRNA are shown in Figure 1(b). The expression level of *T2R7* mRNA in 5 mM chlorpheniramine maleate solution was significantly increased, compared with other lower concentrations (*P* < 0.05), while there were no significant differences observed between chlorpheniramine solutions (1, 0.1, 10^−3^, and 10^−5^ mM) and 50 mM KCL solution (*P* > 0.05).

Interestingly, the relative mRNA abundance of *T2R7* of chicken tongue tissue was remarkably different in all different concentrations of erythromycin solutions and 50 mM KCL solution (Figure 1(c)). We found that *T2R7* mRNA expression in 5 mM erythromycin solution was significantly higher than that in other solutions (*P* < 0.05). The fold changes of 1, 0.1, and 10^−3^ mM erythromycin solutions were significantly higher than those of 50 mM KCL solutions (*P* < 0.05), but there was no difference between erythromycin solutions (*P* > 0.05). The difference between the 10^−5^ mM erythromycin solution and 50 mM KCL solution was not significant (*P* > 0.05). However, the relative expression levels of *T2R7* mRNA were not significantly different between the different concentration gradients of benzoin (Figure 1(d)), chloramphenicol (Figure 1(e)), quinine (Figure 1(f)), parthenolide (Figure 1(g)), and 50 mM KCL solutions (*P* > 0.05).

### 3.2. Effect of Different Bitter Compounds on the Expression of *PLCβ2* mRNA in *cT2R7*/pDisplay-G*α*16/gust44/pcDNA3.1 (+) Cells

**Figure 2:**
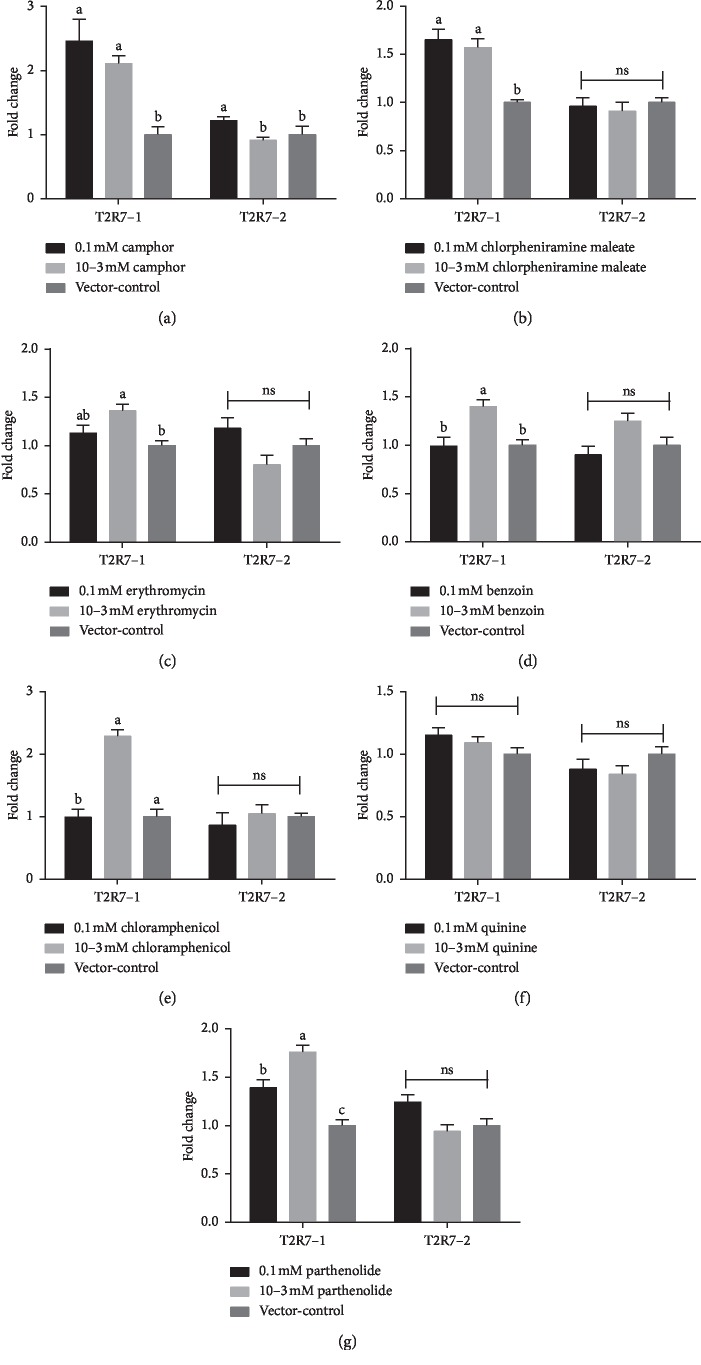
Relative expression levels of *PLCβ2* mRNA in *cT2R7*-1/pDisplay-G*α*16/gust44/pcDNA3.1 (+) and *cT2R7*-2/pDisplay-G*α*16/gust44/pcDNA3.1 (+) cells stimulated by different concentrations of (a) camphor, (b) chlorpheniramine maleate, (c) erythromycin, (d) benzoin, (e) chloramphenicol, (f) quinine, and (g) parthenolide, as determined by real-time PCR. The relative gene expression was measured by qPCR and normalized to GAPDH, and the vector-control was the negative control. Each bar represents the mean ± SE of the results from 2 to 3 independent experiments performed in triplicate. Different small letters on the bar indicate significant differences at *P* < 0.05.

We determined two concentration solutions (0.1 mM and 10^−3^ mM) with reference to the results shown in Figure 2. We found that after exposure to 0.1 and 10^−3^ mM camphor solutions the relative *PLCβ2* mRNA expression was significantly higher than that of the vector-control in *cT2R7-1*/pDisplay-G*α*16/gust44/pcDNA3.1 (+) cells (*P* < 0.05) (Figure 2(a)). On the other hand, the stimulus of 0.1 mM camphor solution increased expression level of *PLCβ2* mRNA significantly than that of the 10^−3^ mM camphor solution and vector-control in *cT2R7-2*/pDisplay-G*α*16/gust44/pcDNA3.1 (+) cells (*P* < 0.05) (Figure 2(a)). Likewise, in *cT2R7-1*/pDisplay-G*α*16/gust44/pcDNA3.1 (+) cells, the relative *PLCβ2* mRNA expression after exposure to stimuli 0.1 and 10^−3^ mM chlorpheniramine maleate solutions was significantly higher than that of the vector-control (*P* < 0.05), whereas there was no remarkable difference measured between chlorpheniramine maleate solutions and the vector-control in *cT2R7-2*/pDisplay-G*α*16/gust44/pcDNA3.1 (+) cells (*P* > 0.05) (Figure 2(b)). The stimulus of 10^−3^ mM erythromycin solution remarkably increased relative *PLCβ2* mRNA abundance than that of the 0.1 mM erythromycin solution and vector-control (*P* < 0.05), but the 0.1 mM erythromycin solution showed no significant difference with the other two treatments in *cT2R7*-1/pDisplay-G*α*16/gust44/pcDNA3.1 (+) cells (*P* > 0.05). However, there was no significant difference in fold changes among the three treatment groups *in cT2R7*-2/pDisplay-G*α*16/gust44/pcDNA3.1 (+) cells (*P* > 0.05) (Figure 2(c)).

Furthermore, after exposure with chemical compounds benzoin (Figure 2(d)) and chloramphenicol (Figure 2(e)), a significant increase occurred in the relative expression level of *PLCβ2* mRNA in the 10^−3^ mM solution compared with that in the other concentration in *cT2R7*-1/pDisplay-G*α*16/gust44/pcDNA3.1 (+) cells (*P* < 0.05), whereas the all three treatment groups were not significantly different in *cT2R7*-2/pDisplay-G*α*16/gust44/pcDNA3.1 (+) cells (*P* > 0.05). As illustrated in Figure 2(f), the relative expression levels of *PLCβ2* mRNA were not significantly influenced by different concentrations of quinine compared with the vector-control (*P* > 0.05). The change in relative expression levels of *PLCβ2* mRNA caused by parthenolide is summarized in Figure 2(g). The stimulus of 10^−3^ mM solution had a significantly higher relative expression level than that of the 0.1 mM solution and vector-control, and the level in the 0.1 mM solution was significantly higher than that of the vector-control in *cT2R7*-1/pDisplay-G*α*16/gust44/pcDNA3.1 (+) cells (*P* < 0.05). By contrast, all the three treatment groups were not significantly different in *cT2R7*-2/pDisplay-G*α*16/gust44/pcDNA3.1 (+) cells (*P* > 0.05).

### 3.3. Effect of Different Bitter Compounds on the Expression of *ITPR3* mRNA in *cT2R7*/pDisplay-G*α*16/gust44/pcDNA3.1 (+) Cells

**Figure 3:**
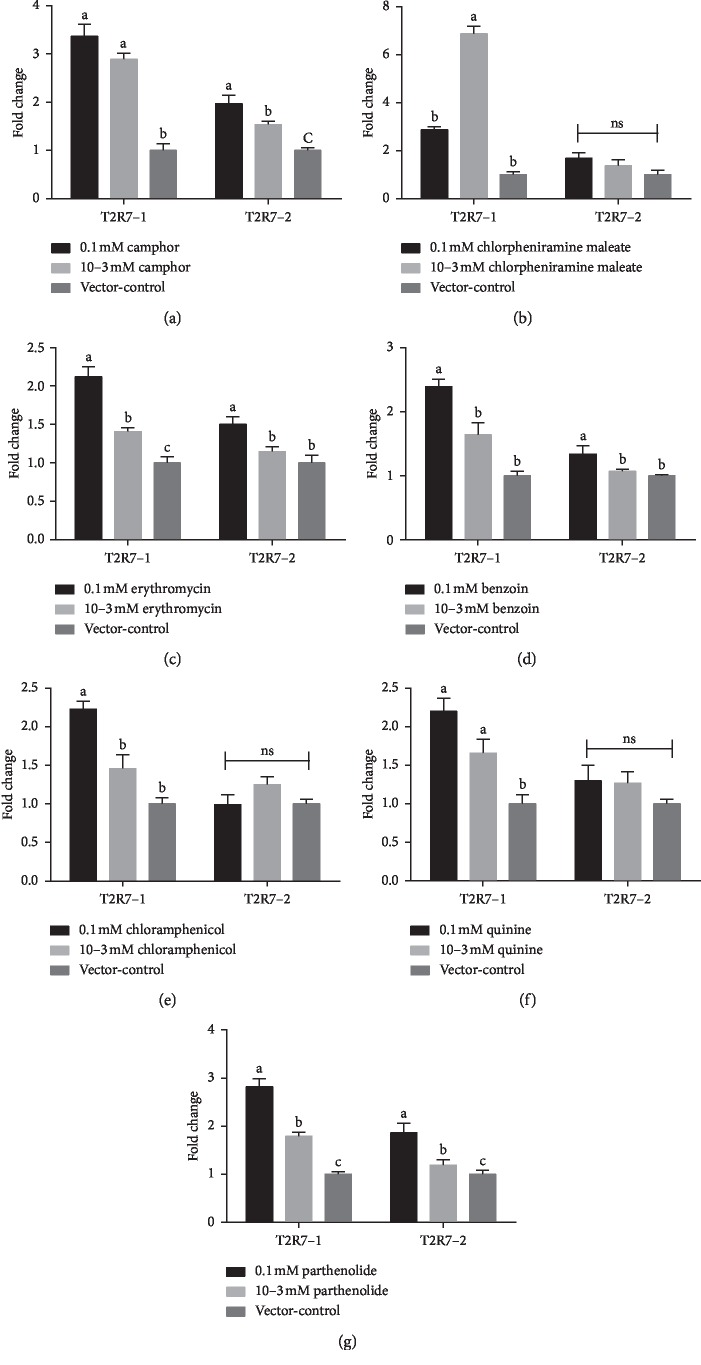
Relative expression levels of *ITPR3* mRNA in *cT2R7*-1/pDisplay-G*α*16/gust44/pcDNA3.1 (+) and *cT2R7*-2/pDisplay-G*α*16/gust44/pcDNA3.1 (+) cells stimulated by different concentrations of (a) camphor, (b) chlorpheniramine maleate, (c) erythromycin, (d) benzoin, (e) chloramphenicol, (f) quinine, and (g) parthenolide, as determined by real-time PCR. The relative gene expression was measured by qPCR and normalized to GAPDH, and the vector-control was the negative control. Each bar represents the mean ± SE of the results from 2 to 3 independent experiments performed in triplicate. Different small letters on the bar indicate significant differences at *P* < 0.05.

In addition to the above analysis, *cT2R7*/pDisplay-G*α*16/gust44/pcDNA3.1 (+) cells were treated with 0.1 mM and 10^−3^ mM compound solutions, and the expression of *ITPR3* mRNA was measured by real-time PCR. The relative expression level is summarized in Figure 3. After exposure with camphor (Figure 3(a)) and quinine (Figure 3(f)) compounds, a significant increase occurred in the relative expression levels of *ITPR3* mRNA in the 0.1 and 10^−3^ mM solutions compared with that of the vector-control in *cT2R7*-1/pDisplay-G*α*16/gust44/pcDNA3.1 (+) cells (*P* < 0.05). The expression level in the stimulus of 10^−3^ mM camphor solution was significantly higher than that in the 0.1 mM camphor solution and vector-control, and the expression level of the 0.1 mM solution was significantly higher than that of the vector-control in *cT2R7*-2/pDisplay-G*α*16/gust44/pcDNA3.1 (+) cells (*P* < 0.05). On the other hand, there was no significant difference detected among the three treatment groups for quinine (*P* > 0.05). After exposure with chlorpheniramine maleate, the stimuli of 10^−3^ mM solution was significantly increased in the relative expression of *ITPR3* mRNA compared with that of 0.1 mM and vector-control in *cT2R7*-1/pDisplay-G*α*16/gust44/pcDNA3.1 (+) cells (*P* < 0.05) (Figure 3(b)); however, no significant differences were found in expression level of *ITPR3* mRNA between these stimuli in *cT2R7*-2/pDisplay-G*α*16/gust44/pcDNA3.1 (+) cells (*P* > 0.05). After exposure with erythromycin (Figure 3(c)), benzoin (Figure 3(d)), and chloramphenicol (Figure 3(e)) compounds, a significant increase occurred in the relative expression level with the stimulus of 0.1 mM solution compared with that in the other concentrations (*P* < 0.05). Moreover, the level in the 10^−3^ mM erythromycin solution was significantly higher than that in the vector-control in *cT2R7*-1/pDisplay- G*α*16/gust44/pcDNA3.1 (+) cells (*P* < 0.05). By contrast, all the three treatment groups for chloramphenicol were not significantly different in *cT2R7*-2/pDisplay- G*α*16/gust44/pcDNA3.1 (+) cells (*P* > 0.05). Similar patterns of expression level were observed in both the *cT2R7*-1/pDisplay-G*α*16/gust44/pcDNA3.1 (+) and *cT2R7*-2/pDisplay-G*α*16/gust44/pcDNA3.1 (+) cells after exposure with parthenolide solution. We determined that the 10^−3^ mM parthenolide solution had a significantly higher relative expression level than that of the 0.1 mM solution and vector-control (*P* < 0.05). Further, the level of mRNA expression in the 0.1 mM parthenolide solution was significantly higher than that of the vector-control in *cT2R7*-1/pDisplay-G*α*16/gust44/pcDNA3.1 (+) cells (*P* < 0.05) (Figure 3(g)).

## 4. Discussion

Tibetan chickens living in Tibet plateau have adapted to the special environment and developed unique genetic predispositions and dietary habits [32]. Jiuyuan black-chickens used in the study are important as egg layers. It has been demonstrated that the broiler chickens (broiler-type) are more sensitive to bitter taste than White Leghorn (layer-type) [33]. Previously, researchers found a relationship between the number of taste buds and taste sensitivity [33, 34]. Furthermore, optimizing the consumption of balanced diets plays a central role in the welfare, development, health, and productivity of animals, especially when raised or preserved in captivity [35].

In this study, we ultimately explore the sensibility of taste receptor proteins to bitter compounds in order to ascertain whether chickens are sensitive to several bitter compounds with concentrations gradient. Significant changes in mRNA expression of *T2R7* were found in a dose-dependent fashion after induction with camphor, chlorpheniramine maleate (*P* < 0.05), and erythromycin solutions, but there was no significant difference found between the different concentrations in the other four compounds (*P* > 0.05). These results implied that the sensitivity of chickens to bitter compounds (camphor, chlorpheniramine maleate, and erythromycin) can be determined by the relative expression of *T2R7* mRNA in tongue tissues. The postnatal administration of the bitter tasting quinine to chicks increases the mRNA expression level of *T2R1* and *T2R7* genes in the chicken's tongue [25], and four chemical substances chloramphenicol, chlorpheniramine, diphenidol, and quinine sulfate activate all three bitter taste receptors in chickens [23]. However, the expression of *T2R7* mRNA was not related to the intake of quinine and chloramphenicol at different concentrations. Recent behavioral experiments reveal that baby chicks have a greater aversion to salt and sour taste qualities than in adults [36]. A latest research on chickens shows that younger chicks are more sensitive to bitter compounds compared with older chicks [37]. Previously, it was reported that NaCl solutions of 85 mM and 100 mM are the preferred solutions for chickens, while chickens refused to accept NaCl solutions of 250 mM or higher concentration due to aversive taste [38]. In this study, we used Jiuyuan black-chickens at age of 120 days, which were fed the normal commercial feed formulation. Thus, we speculate that the age of chickens resulted in a reduction in their sensitivity to bitter compounds. Additionally, normal commercial feed does not contain strongly bitter substances [37]; therefore, the bitter taste experience of these chickens was not extensive under the experimental conditions of this study. Moreover, the long-term feed formulation and highly selective breeding of farm-raised chickens expose them to less toxic bitter substances that exist in nature. Therefore, we speculate that this series of artificial interventions resulted in the degeneration of the *T2R7* gene during the long-term breeding process of chicken. Certainly, additional studies are necessary to confirm this hypothesis.

Furthermore, quinine stimulation elevated the mRNA level of *ITPR3* without affecting the *T2R7* and *PLCβ2* mRNA levels, and these results are consistent with earlier studies which show that chickens are less sensitive to quinine compared to the other bitter compounds [30, 39]. Our findings are in accordance with a recent study which demonstrated that *IP3R3*-mediated Ca^2+^ flux is strongly inhibited by quinine using heterologous systems and/or cell lines [40]. The gustducin activates phospholipase C (*PLCβ2*) and catalyzes phosphatidylinositol phosphate to produce the second messengers inositol 1,4,5 trisphosphate (*IP*_*3*_) and diacylglycerol (*DAG*), leading to calcium release [41, 42]. A prior study using knockout mice reported that the elimination of *PLCβ2*, *TrpM5*, or *IP*_*3*_ receptor proteins leads to a reduction or complete loss of sensitivity to bitter, sweet, or umami taste suggesting that these taste sensations strongly favor a conserved, streamline signaling cascade [43]. Thus, a bitter substance can induce the activation of the bitter receptor protein and enhance the relative *PLCβ2* and *ITPR3* mRNA expression. These results provide favorable evidence for reducing the sensitivity of different bitter compounds to bitter taste, although further research is required to confirm this hypothesis.

Bitter compounds elicit innate aversive response across species, which is typically considered to prevent the ingestion of toxic substances [44, 45]. Hirai et al. established correlation between patients' taste sensitivity, perception, and its mRNA expression, which increases the expression of taste receptor genes linked to phantogeusia pathogenesis by enhancing bitter taste sensation [46]. We screened several bitter compounds identified through inquiry of the BitterDB probe (http://bitterdb.agri.huji.ac.il/bitterdb/, [47]) to explore the relative mRNA expression levels in downstream signaling genes (*PLCβ2* and *ITPR3*) of chicken. Previous reports have described *IP3R3* as the dominant form of the IP3 receptor which plays an important role in bitter taste transduction [48, 49]. In addition, knocking out of *PLCβ2* and *ITPR3* reduces the ability of mice to recognize most bitter compounds [50]. We found that the relative mRNA expression levels of *PLCβ2* and *ITPR3* in *cT2R7-1*/pDisplay-G*α*16/gust44/pcDNA3.1 (+) cells induced by different bitter compounds, such as 0.1 and 10^−3^ mM camphor, 10^−3^ mM chlorpheniramine maleate, 10^−3^ mM erythromycin, 10^−3^ mM quinine, and 0.1 mM and 10^−3^ mM parthenolide, were significantly higher than that of the vector-control (*P* < 0.05). Previously, *PLCβ2* and *ITPR3* were identified as the key genes in the signal transduction pathway of bitter taste [49–51]. Bitter taste signaling is believed to be complex, and the pathways that transduce the taste response to bitter compounds lead to cation channel opening, depolarization of the taste receptor cells (TRCs), and subsequent neurotransmitter release [50]. In particular, chemical bitter compounds (quinine, garlic oil, almond oil, clove oil, and magnesium chloride) play a crucial role in reducing feather pecking incidence in laying hens [52–54].

By contrast, the mRNA expression of *PLCβ2* and *ITPR3* in *cT2R7*-2/pDisplay-G*α*16/gust44/pcDNA3.1 (+) cells stimulated by 0.1 mM camphor was significantly higher than that of the vector-control (*P* < 0.05). Primarily, chickens appear to have an acute sense of taste allowing the discrimination of five primary tastes including fatty, umami, salty, sour, and bitter [55]. Interestingly, dietary preferences are suppressed when the tongue becomes paralyzed, suggesting the role of olfaction in dietary fat preferences [56]. Meanwhile, T2Rs have been identified in nonoral tissues, including the respiratory tract, the gut, the spleen, the lung, the heart, and bursa Fabricius, suggesting its involvement in other physiological functions such as appetite regulation, innate immunity, and internal organs, besides taste [57, 58]. In addition, preferences for a balanced diet containing synthetic AA (potentially related to umami taste) suggest that lysine, methionine, and tryptophan in a diet increase feed intake [59]. It has been reported that PKD1L3 and PKD2L1 ion channels function as a likely candidate for a mammalian sour taste receptor related to the generation of acidic taste [60]. Bitter taste receptor harbors multiple single nucleotide polymorphisms (SNPs) in the coding region, which are associated with dietary preferences, metabolic traits, and body mass index [61].

Our results reveal that bitter taste receptor (T2R7) is sensitive to different concentrations of bitter compounds and *T2R7-1* (corresponding to the unique haplotype of the Tibetan chicken) had higher sensitivity to bitter compounds compared with that of *T2R7-2* (corresponding to the unique haplotype of the Jiuyuan black-chicken). Similarly, recent studies have shown an important link between the haplotypes and the ability to identify bitter compounds [62]. They showed that the sequence variants of *T2R38* gene had a direct influence on phenylthiocarbamide (PTC) taste sensitivity, in which the haplotype PAV had greater PTC sensitivity compared with that of the haplotype AVI. In addition, Tibetan chickens raised outside cage may absorb or detect more natural bitter substances than Jiuyuan black-chickens bred in cages. The long-term feed formulation and highly selective breeding may reduce the sensitivity of Jiuyuan black-chickens to bitter substances. Overall, chicken chemosensory research has been applied to chicken initial choice of feed and the level of feed consumption, which is critical to the understanding of molecular mechanisms of chicken taste.

## 5. Conclusions

In summary, we evaluated the sensitivity of bitter taste receptor (T2R7) and its downstream signaling molecule on synthetic bitter compounds for chicken. The bitter taste receptor T2R7 was found sensitive to different concentrations of bitter compounds. Furthermore, bitter taste receptor T2R7-1 (corresponding to the unique haplotype of the Tibetan chicken) has higher sensitivity to bitter compounds compared with that of T2R7-2. Our results will be helpful in the enhancement of chicken feed efficiency as well as beneficial to future taste sensation research in context of improving feeding strategies among Aves.

## Figures and Tables

**Table 1:** Polymorphic sites identified in the coding region sequence of *T2R7-1* and *T2R7-2* gene in chicken.

Nucleotide position	KT377158 (*T2R7-1*)	KT377161 (*T2R7-2*)	Mutation type	Amino acid change
280	A	C	Transversion	Met > Leu
766	A	T	Transversion	Thr > Ser
802	G	A	Transition	Val > Met

**Table 2:** Detailed information of the primers used in real-time PCR analysis.

Primer	Forward primer	Reverse primer	Fragment length (bp)	Annealing temperature (°C)	Accession numbers
*GAPDH-1*	CCAGAACATCATCCCAGCGTC	ACGGCAGGTCAGGTCAACAA	136	60.0	NM_204305.1
*GAPDH-2*	CTTTGGTATCGTGGAAGGACTC	GTAGAGGCAGGGATGATGTTCT	132	60.0	NM_002046
*T2R7*	TTCAGGCACCATTTCTTCATCTAC	TGGGGCTGGTTCTGTTCTCT	142	60.0	NM_001080719.1
*PLCβ2*	AAGATGCCCAAGAGCCAGAAG	GGAGACGAAGTTGTGGAAGGTG	132	56.4	NM_001284299.1
*ITPR3*	TCCTGTTCTTCTTCATCGTCATCA	TTGTTATCAAACTTGTCCCTCTCCA	160	60.0	NM_002224.3

## Data Availability

All data are available within the article or available from the authors upon request.
